# Omicron subvariants illustrate reduced respiratory tissue penetration, cell damage and inflammatory responses in human airway epithelia

**DOI:** 10.3389/fimmu.2023.1258268

**Published:** 2023-10-17

**Authors:** Viktoria Zaderer, Hussam Abd El Halim, Anna-Lena Wyremblewsky, Gaia Lupoli, Christopher Dächert, Maximilian Muenchhoff, Alexander Graf, Helmut Blum, Cornelia Lass-Flörl, Oliver T. Keppler, Lukas A. Huber, Wilfried Posch, Doris Wilflingseder

**Affiliations:** ^1^Institute of Hygiene and Medical Microbiology, Medical University of Innsbruck, Innsbruck, Austria; ^2^Virology, Max von Pettenkofer Institute and Gene Center, Ludwig-Maximilians-Universität (LMU), Munich, Germany; ^3^Laboratory for Functional Genome Analysis, Gene Center, Ludwig-Maximilians-Universität (LMU), Munich, Germany; ^4^Institute of Cell Biology, Biocenter, Medical University of Innsbruck, Innsbruck, Austria; ^5^ADSI - Austrian Drug Screening Institute GmbH, Innsbruck, Austria

**Keywords:** SARS-CoV-2, VOCs, delta, omicron, epithelial barrier model

## Abstract

**Introduction:**

To explore whether the reported lower pathogenicity in infected individuals of variant of concern (VoC) Omicron and its current subvariants compared to VoC Delta may be related to fundamental differences in the initial virus-tissue interaction, we assessed their ability to penetrate, replicate and cause damage in a human 3D respiratory model.

**Methods:**

For this, we used TEER measurements, real-time PCR, LDH, cytokine and complex confocal imaging analyses.

**Results and discussion:**

We observed that Delta readily penetrated deep into the respiratory epithelium and this was associated with major tissue destruction, high LDH activity, high viral loads and pronounced innate immune activation as observed by intrinsic C3 activation and IL-6 release at infection sites. In contrast, Omicron subvariants BA.5, BQ.1.1 and BF7 remained superficially in the mucosal layer resulting merely in outward-directed destruction of cells, maintenance of epithelial integrity, minimal LDH activity and low basolateral release of virus at infection sites, as well as significantly smaller areas of complement activation and lower IL-6 secretion. Interestingly, also within Omicron subvariants differences were observed with newer Omicron subvariants BQ.1.1 and BF.7 illustrating significantly reduced viral loads, IL-6 release and LDH activity compared to BA.5. Our data indicate that earliest interaction events after SARS-CoV-2 transmission may have a role in shaping disease severity.

## Highlights

- Delta penetrates deep into the respiratory epithelium and is associated with tissue destruction, cellular stress, innate immune activation and high viral loads.
- Omicron subvariants remain apically distributed in respiratory tissues and are associated with outward directed cell destruction, low cell stress and innate immune activation and low basolateral virus release.

## Introduction

Novel SARS-CoV-2 variants of concern (VoC) rapidly emerge. With multiple mutations in its receptor-binding domain (RBD), Omicron (B.1.1.529 with subvariants BA.1, BA.2, BA.4, BA.5, BQ1.1, BF7, XBB.1.5) is entirely different from the former dominant Delta variant in terms of its contagiousness, evasion of pre-existing antibodies, and evidence of milder disease progression (1–5). Rapid increases in cases, reinfections as well as vaccine breakthrough infections with the Omicron variants have been reported. Nevertheless, there are several lines of evidence world-wide that Omicron causes a less severe form of COVID-19 with less hospitalizations of adults relative to previous variants such as Delta (B.1.617.2) (1, 6). In January 2022, the two Omicron lineages, BA.4 and BA.5, appeared in South Africa and over the last months several other Omicron subvariants (BQ.1, BQ.1.1, XBB.1.5, BF7) emerged (3). While BQ.1 and BQ.1.1 evolved from BA.5, recombination of two BA.2 lineages resulted in XBB subvariants (7). There is evidence from *in vitro* data with the Omicron variants that the virus itself showed similar replication for Omicron and Delta in human nasal epithelia, while in lung and gut cells the Omicron spike protein was less efficiently cleaved and illustrated lower replication (8). Significant replication defects of Omicron BA.1 were measured relative to Delta in cells where TMPRSS2, needed for cell-cell fusion, was present (8). The rapid displacement of Delta by the less pathogenic Omicron variant as well as the rapid evolution of Omicron subvariants with a broad array of mutations in the spike protein (7) call for a more detailed and comparative characterization of what happens in human target cells upon virus entry and host cell defense. Earlier, a robust engagement of complement concomitant with excessive pro-inflammatory cytokine induction was detected following SARS-CoV-2 infection of highly differentiated, pseudostratified human airway epithelia (HAE) from upper and lower respiratory tract (9). Here, we describe that the Omicron variants BA.1, BA.2, BA.5 and also the newer subvariants BQ.1.1 and BF7 are differently handled within highly differentiated, pseudostratified respiratory epithelia compared to Delta in terms of tissue penetration and cell destruction, intrinsic complement and IL-6 activation as well as LDH and virus release.

## Materials and methods

### Human airway epithelial cell culture

Normal human bronchial epithelial (NHBE, Lonza, cat# CC-2540 S, upper respiratory tract) are available in our laboratory and routinely cultured in air liquid interface (ALI) as described (9–11). Briefly, cells were cultured as a monolayer for 2–4 days until they reached 80% confluence. Cells were detached and seeded onto GrowDexT (UPM)-coated 0.33 cm^2^ porous (0.4 μm) polyester membrane inserts with a seeding density of 1 × 10^5^ cells per Transwell (Costar, Corning, New York, NY, USA). The cells were grown to near confluence in submerged culture for 3 days in specific epithelial cell growth medium according to the manufacturer´s instructions (Stemcell™). Cultures were maintained in a humidified atmosphere with 5% CO2 at 37 °C and then transferred to ALI culture for another four weeks until cells were fully differentiated. The epithelium was expanded and differentiated using airway media from Stemcell™. The number of days in development was designated relative to initiation of ALI culture, corresponding to day 0.

### Vero cells

VeroE6/TMPRSS2/ACE2 is an engineered VeroE6 cell line expressing high levels of TMPRSS2 and ACE2 and highly susceptible to SARS-CoV-2 infection. This cell line was used to expand characterized BA.1 and BA.4/5 viruses from patient isolates and to perform plaque assays to test infectivity. The cell line was obtained via the CFAR (NIBSC) and is described in (9).

### Transepithelial electrical resistance measurement

TEER values were measured using EVOM volt-ohm-meter with STX-2 chopstick electrodes (World Precision Instruments, Stevenage, UK) as described (9). Briefly, for measurements, 100 µl and 700 µl of medium were added to the apical and basolateral chambers, respectively, and cells were allowed to equilibrate before TEER was measured. TEER values reported were corrected for the resistance and surface area of the Transwell filters.

### Staining and high content screening

To visualize SARS-CoV-2 infection in monolayers and 3D tissue models, cells were infected with clinical specimen of SARS-CoV-2 Delta and Omicron variants and analyzed for characteristic markers in infection experiments on day 2 post infection (2 dpi). After SARS-CoV-2 exposure, 3D cell cultures were fixed with 4% paraformaldehyde. Intracellular staining was performed using 1x Intracellular Staining Permeabilization Wash Buffer (10x; BioLegend, San Diego, CA, USA). Cells were stained using phalloidin-Alexa647 (Thermofisher Scientific, Waltham, MA, USA), nuclei using Hoechst (Cell Signaling Technologies, Danvers, MA, USA), and complement C3 using a C3-FITC antibody (Agilent Technologies, Santa Clara, CA, USA). Intracellular SARS-CoV-2 was detected using Alexa594-labeled SARS-CoV-2 antibodies against N (Sino Biological, Beijing, China). The Alexa594-labeling kit was purchased from Abcam, Cambridge, UK. After staining, 3D cultures were mounted in Mowiol. To study these complex models using primary cell cultured in 3D and to generate detailed phenotypic fingerprints for deeper biological insights in a high throughput manner, the Operetta CLS System (PerkinElmer, Waltham, MA, USA) was applied. Spot analyses and absolute quantification for SARS-CoV-2-containing cells, Harmony™ Software was performed in more than 2000 cells per condition, since in five independent experiments at least 400 cells defined by nuclear stain (Höchst) were analyzed.

### Real-time RT-PCR for absolute quantification of SARS-CoV-2

SARS-CoV-2 RNA was extracted using FavorPrep Viral RNA Mini Kit, according to manufacturer’s instructions (Favorgen Europe, cat# FAVRE 96004, Austria). Sequences specific to 2 distinct regions of the Nucleocapsid (N) gene, N1 and N2, and for the detection of a human housekeeping gene, Ribonuclease P, were used. Single target assays of all 3 targets were performed in combination with the Luna Universal Probe One-Step RT-qPCR Kit (New England Biolabs, cat# E3006, Germany). For absolute quantification using the standard curve method, SARS-CoV-2 RNA was obtained as a PCR standard control from the National Institute for Biological Standards and Control, UK. All runs were performed on a Bio-Rad CFX 96 instrument and analyzed by the Bio-Rad CFX Maestro 1.1 software (Bio-Rad, Germany).

### Viruses

Clinical specimens for SARS-CoV-2 VoC Delta and Omicron subvariants (BA.1, BA.2, BA.5, BQ1.1, BF7) from sequenced COVID-19 positive swabs were propagated and subsequently used to infect cells at a MOI of 0.0025. All experiments, where we used live virus strains (infection, plaque assays) were performed under BSL3 conditions.

### Plaque assay

VeroE6/ACE2/TMPRSS2 cells were inoculated with serial dilutions of 2 dpi subnatants from Omicron subvariant- or Delta-infected HAE cells for 1h at 37°C/5%CO_2_. Inoculate was replaced with culture medium containing 1.5% carboxymethylcellulose and incubated for 3 days at 37°C/5% CO_2_ before plaque visualization and counting as described (12).

### Statistical analysis

Statistical analysis of differences in infection levels, TEER values, or cytokine production was performed utilizing the GraphPad prism software and using OneWay ANOVA with Tukey´s posttest.

### Ethics statement

Written informed consent was obtained from all donors of leftover nasopharyngeal/oropharyngeal specimens and EDTA blood by the participating clinics. The Ethics Committee of the Medical University of Innsbruck (ECS1166/2020) approved the use of anonymized leftover specimens of COVID-19 patients for scientific purposes.

## Results

### Delta penetrates deep into the pseudostratified, columnar tracheal epithelium, while Omicron variants remain superficial

**Figure 1:**
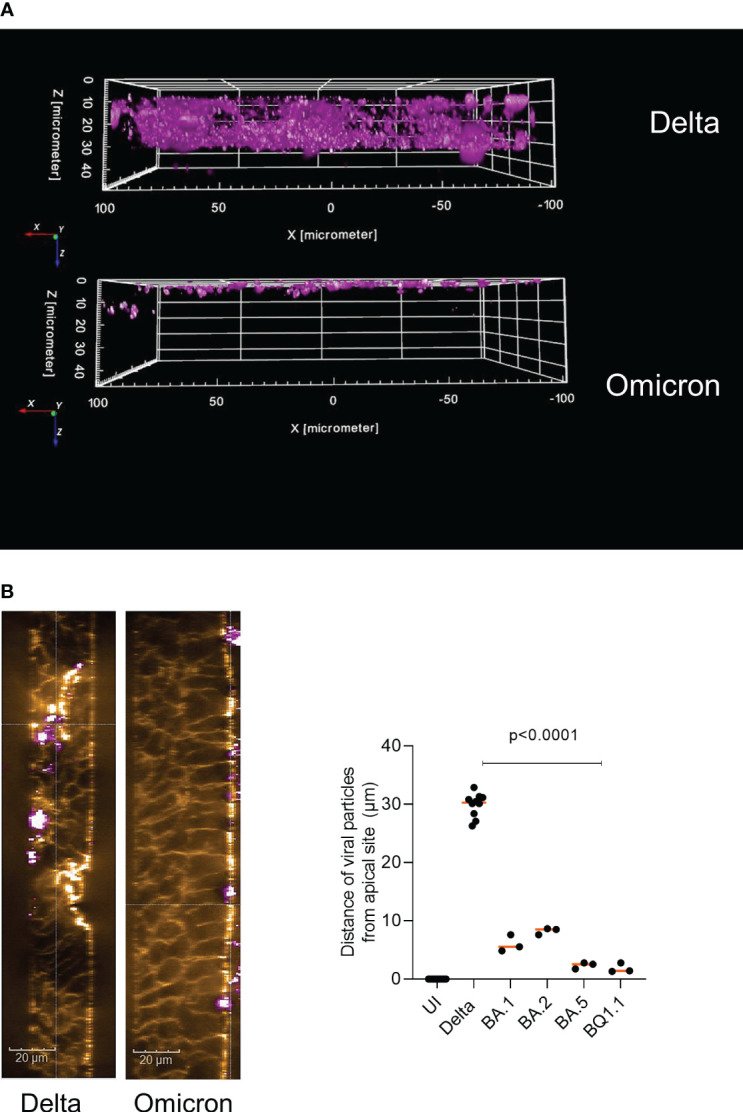
Delta penetrates deep into HAE cells compared to apical distribution of Omicron. Pseudostratified epithelia were infected by apical addition of SARS-CoV-2 Delta or Omicron subvariants BA.1, BA.2, BA.5 or BQ1.1 for 48h and immunofluorescence analyses were performed to stain for virus (pink), actin (orange), C3 (green) and nuclei (blue). **(A)** 3D image analyses of virus penetration into the pseudostratified HAE model generated using the Harmony™ software (Perkin Elmer). In panel A, the virus signal in Delta vs. Omicron infected tissues is depicted, while in **(B)** an XYZ-analyses was performed, illustrating the ZY-axis and phalloidin (orange) and virus (pink) staining (left), scale bars 20µm. The experiment was repeated 8 to 10 times using Delta and various Omicron subvariants and distance was statistically determined by one-way ANOVA with Tukey´s multiple comparisons test. Each dot represents an independent experiment and means are depicted in orange (right).

First, we analyzed the localization of Delta and Omicron-infected epithelial cultures by immunofluorescence followed by 3D analysis. We noticed that Delta distributed over the entire width of the epithelium and localized very close to the basement membrane (**Figure 1A**, upper panel, **Supplementary Figure 1**, middle, right panel). In contrast, all Omicron subvariants tested in immunofluorescence analyses (BA.1, BA.2, BA.5, BQ1.1) remained apically distributed and did not dig that deep into the pseudostratified columnar epithelium (**Figure 1A**, lower panel, **Supplementary Figure 1**, Omicron (BA.5), right panel). Next, we analyzed the infected tissue models in more detail using phalloidin staining and performing XYZ analysis. The widespread dissemination of the Delta variant reaching the basement membrane became even more apparent, while Omicron only associated with the apical plasma membrane, but did not penetrate into the tissue (**Figure 1B**, left). When measuring the distance of viral particles from the apical site in multiple samples, a highly significant (p<0.0001) difference between Delta and Omicron subvariants BA.1, BA.2, BA.5 and BQ1.1 was illustrated, with Delta diving deep (mean 30 µm) into the columnar, pseudostratified epithelium and Omicron remaining on the surface of the epithelium (mean 3.5 µm) (**Figure 1B**, right). Our data indicate that Delta is more likely able to penetrate the tissue, while Omicron stays superficially.

### Epithelial integrity of HAE cultures is retained upon Omicron infection, while interrupted by Delta

**Figure 2:**
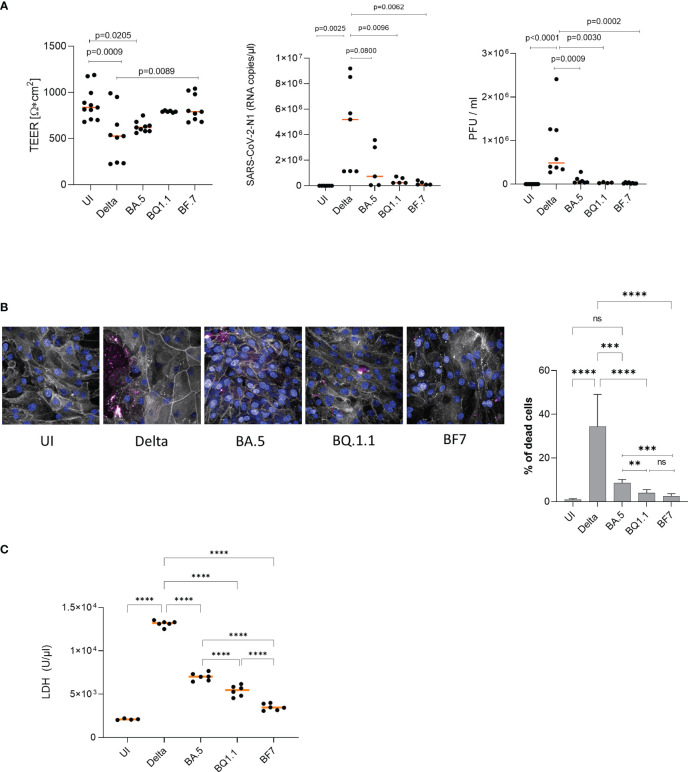
(**A**, left) Epithelial integrity is significantly disrupted in Delta versus Omicron. TEER was measured on 2 dpi (left) using a EVOM volt-ohm-meter. TEER in Ω/cm^2^ was determined for all conditions (UI, Delta, Omicron subvariants BA.5, BQ1.1, BF.7) and plotted on a dot graph. Dots represent infection experiments of pseudostratified epithelia using Delta or Omicron subvariants BA.5, BQ1.1 or BF.7 measured in triplicates. Statistical significance was calculated using One-way ANOVA with Tukey´s multiple comparisons test, means are depicted in orange. (**A**, middle, right) Viral load and infectivity are significantly higher in Delta versus Omicron. (**A**, middle) Viral load was determined by absolute quantification using real-time RT-PCR and a SARS-CoV-2 N standard. While Delta-infected epithelia depicted a high basolateral viral load, HAE infected with Omicron subvariants illustrated low-level infection, which corresponded to (**A**, right) the infectivity of released viruses as measured by plaque assay on VeroE6/ACE2/TMPRSS2-expressing cells. **(B)** Delta mediates higher cell death in HAE compared to Omicron subvariants BA.5, BQ1.1 and BF7. Immunofluorescence staining of HAE cells infected for 2 days with Delta or the indicated Omicron subvariants was performed using phalloidin (orange), Höchst (blue) and a live/dead cell staining kit (red) as described. % of dead cells were calculated using the Harmony™ software and one-way ANOVA with Tukey´s multiple comparisons test. **(C)** LDH activity is significantly higher in Delta versus Omicron. Cytotoxicity was analyzed using the Cytotoxicity Detection Kit (LDH) from Roche according to the manufacturer´s instructions (Merck, cat# 1164493001, Austria). This kit is based on measuring LDH activity released from damaged cells. Treatment of 3D human respiratory tissue models with Delta or Omicron subvariants BA.5, BQ1.1 or BF7 revealed a significantly higher LDH activity in cells infected with Delta- versus all tested Omicron subvariants. The newest subvariants BQ1.1 and BF7 were even significantly lower in their LDH activities compared to the BA.5 subvariant, indicating less cell stress and cytotoxicity. Statistical significance was calculated using One-way ANOVA with Tukey´s multiple comparisons test, means are depicted in orange. **p<0.01; ***p<0.001; ****p<0.0001. ns not significant.

To monitor, if the observed differences in tissue penetration between Delta and Omicron are associated with differences in epithelial integrity, transepithelial electrical resistance (TEER) was measured on 2 days post infection (2 dpi) using a low multiplicity of infection (MOI) of 0.0025 for the virus preparations to avoid too fast tissue destruction and cell death. From here on, only the more recent Omicron subvariants BA.5, BQ1.1, BF.7 are illustrated, since results from BA.1 and BA.2-infected cultures were earlier published by our group (10, 13). Despite low MOI, analyses revealed that upon Delta infection, TEER values of HAE cells significantly dropped compared to mock-treated cells (UI, uninfected) and to cells infected with the Omicron subvariants BQ1.1 and BF.7 (**Figure 2A**, TEER). Omicron BQ1.1- and BF.7-infected cultures were similar in their TEER values relative to UI ones on 2 dpi, while BA.5 showed significantly lower TEER values (**Figure 2A**, TEER). From day 3 post infection on, tissue destruction was also observed using Omicron subvariants, since the Transwell is a closed system and viruses cannot be cleared. Here, we found that epithelial integrity is more likely maintained in Omicron- versus Delta-infected HAE cultures.

### Viral loads are significantly lower in Omicron- versus Delta-infected HAE cultures

In accordance to image analyses and TEER, absolute quantification of viral load from 2 dpi supernatants of differently treated cells revealed a significantly lower SARS-CoV-2-N1 RNA copy number in Omicron subvariant- compared to Delta-infected HAE cultures after infection at a MOI 0.0025 (**Figure 2A**, middle). Here, all Omicron subvariants tested (BA.5, BQ1.1, BF.7) revealed significantly lower viral loads compared to Delta. This resulted in highly significant reduction of viral titers as analyzed by plaque assay of 2 dpi subnatants from infected HAE cells (**Figure 2A**, right). Mock-treated cultures (UI) served as controls. Defective replication and infectivity of Omicron variants compared to Delta within highly differentiated HAE cells were illustrated herein.

### Cell viability is significantly higher in Omicron- versus Delta-infected HAE cultures

Next, we discriminated live and dead cells in cultures infected with either Delta or Omicron BA.5, BQ1.1 or BF7 by using amine-reactive dyes appropriate for fixed samples. Immunofluorescence analyses revealed significantly higher numbers of dead cells upon infection with the Delta variant compared to all Omicron subvariants tested (BA.5, BQ1.1, BF7) (**Figure 2B**, left and right). On 2 dpi, only isolated dead cells were detected in uninfected (UI), or Omicron BQ1.1 or BF7-infected cultures, while BA.5-infected cells demonstrated higher cell death (**Figure 2B**, left, dead cells – red, phalloidin – gray, nuclei – blue). Delta-infected cells showed the highest cell death rates (**Figure 2B**, left). When analyzing percentages of dead cells, between 20 to 50% were detected in Delta-infected cultures, and between 1 to 9% in cell cultures infected with Omicron BA.5, BQ1.1 or BF7 (**Figure 2B**, right). BA.5 illustrated a significantly higher cell death rate compared to the newer emerging subvariants BQ1.1 or BF7 (**Figure 2B**, right). Further image analyses revealed leaky tissue structures with big areas of cell detachment in Delta-infected tissues (**Supplementary Figure 1**, 2^nd^ panel), while Omicron subvariants displayed only superficial cell destruction without porous structures (**Supplementary Figure 1**, 3^rd^ panel). UI cultures served as controls (**Supplementary Figure 1**, 1^st^ panel). An overview of UI, Delta, and Omicron-infected cells and nuclei counts of at least 2000 cells is depicted in **Supplementary Figure 1**, 4^th^ panel. Thus, Delta displays considerably higher cell death of HAE cells compared to all Omicron subvariants tested, and cell death of BA.5-infected cultures is significantly higher compared to BQ1.1- and BF7-exposed cells.

### Delta triggers significantly higher LDH release compared to Omicron

Moreover, we measured LDH activity of HAE cells exposed to Delta versus Omicron subvariants BA.5, BQ.1.1 and BF7. We found that upon infection with Delta, excessive LDH activation was measured that was significantly higher compared to UI, BA.5, BQ.1.1 and BF7 (**Figure 2C**). In accordance to the higher cell destruction observed, the Omicron BA.5 subvariant mediated significantly higher LDH activity compared to BQ.1.1 and BF7, and BF7 exerted lowest LDH activity levels (**Figure 2C**). Thus, the enhanced tissue damage observed with the Delta variant was accompanied by significantly higher LDH activity compared to all Omicron subvariants.

### Intrinsic C3 activation and IL-6 production are induced to significantly lower levels in Omicron- vs. Delta-infected HAE cultures

**Figure 3:**
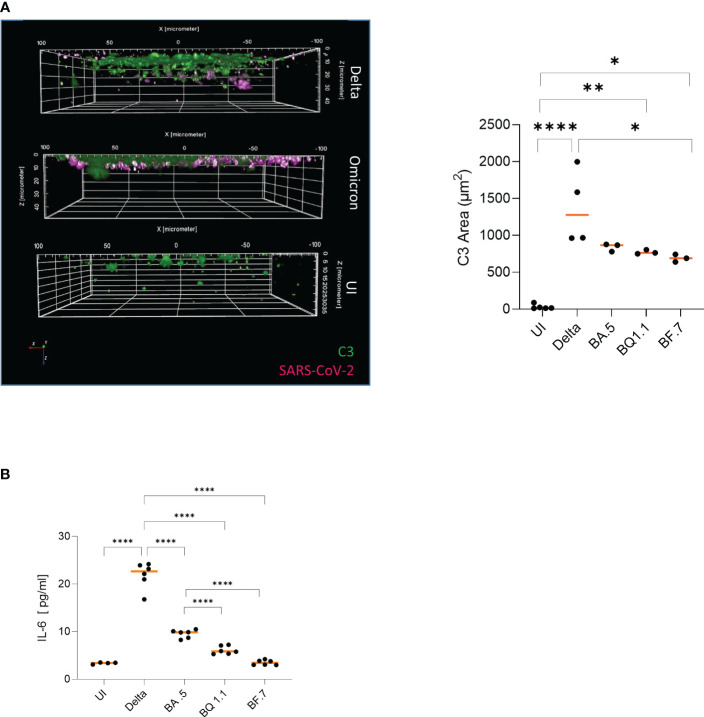
**(A)** Higher local complement C3 is induced in Delta versus Omicron-infected epithelia. Visualization of virus binding (SARS-CoV-2 N, pink) and complement (C3-FITC, green) in SARS-CoV-2 infected 3D pseudostratified epithelia. On 2 dpi, filters were fixed, stained for SARS-CoV-2 N (pink), and complement C3 (green) then analyzed by HCS. Higher IC C3 mobilization was monitored in Delta versus Omicron-infected cultures (right), while no virus and low C3 signals were detected in UI. Areas of C3 production were absolutely quantified from at least three different areas using the Harmony™ software (right). Statistical significances were analyzed with GraphPad Prism software using One-way ANOVA and Tukey´s posttest. As representative image, infection with Omicron subvariant BA.5 is depicted. **(B)** Significantly higher IL-6 is induced in Delta versus Omicron-infected epithelia. Intracellular C3 mobilization was associated with pro-inflammatory cytokine induction like IL-6. Upon infection of HAE cells with in particular Delta or the Omicron subvariant BA.5, significantly higher IL-6 secretion was mediated in contrast to BQ1.1-, BF7- or uninfected cultures. Statistical significances were analyzed with GraphPad Prism software using One-way ANOVA and Tukey´s posttest, means are depicted in orange. * p<0.05; **p<0.01; ****p<0.0001.

Extensive intrinsic C3 linked to excessive secretion of the anaphylatoxin C3a at infection sites was reported in patient samples´ bronchoalveolar lavages (BALs) and from HAE cells upon infection with previous variants of SARS-CoV-2 (9, 14, 15). Thus, we were interested, if the dissimilar processing of Delta and Omicron subvariants within HAE cells is linked to a difference in intracellular C3 and pro-inflammatory cytokine (IL-6) production. 3D analyses of Z-stacks over the entire width of mock-treated, Delta- and Omicron-BA.5-infected HAE cultures illustrated a greater area of C3-positive cells (green) in Delta-infected cultures in the epithelium compared to Omicron (**Figure 3A**; **Supplementary Figure 1**, 2^nd^ and 3^rd^ panel). Despite the significantly lower productive infection, tissue destruction and superficial localization, Omicron mediated a significantly higher intrinsic C3 generation (green) compared to mock-treated cells (**Figure 3A**, Omicron subvariants vs. UI). Nevertheless, intracellular (IC) C3 produced was still substantially reduced in Omicron- versus Delta-infected cultures, independent on the subvariant used. As shown earlier (9), IC C3 production and subsequent C3a release resulted in induction of pro-inflammatory cytokines IL-1a, RANTES, MIP-1β and IL-6 from highly differentiated HAE cells. Analyzing IL-6 following infection with Delta versus Omicron subvariants BA.5, BQ.1.1 and BF7 revealed a significantly higher cytokine secretion in Delta-infected cultures relative to UI or Omicron-infected cultures. Moreover, we found that the BA.5 VOC caused significantly higher IL-6 release compared to the newer subvariants BQ.1.1 or BF.7 (**Figure 3B**), nicely correlating to the inflammatory response induced by the different subvariants. These data reveal that Omicron infection of differentiated HAE cells resulted in substantial C3 generation compared to UI, which is still significantly lower relative to Delta and not sufficient to effectively induce excessive pro-inflammatory responses.

## Conclusions

Here, we analyzed in more detail entry and early events during the infection process of Delta versus Omicron subvariants (BA.1, BA.2, BA.5, BQ.1.1, BF7) in highly differentiated, primary human airway epithelial cells grown at an air-liquid-interphase. These analyses revealed that Delta massively penetrates the pseudostratified human airway epithelium, thereby resulting in excessive tissue destruction, cell death, as well as high inflammatory responses. In contrast, all Omicron subvariants tested remained apically on the airway epithelium causing cell death only superficially, and stress-response and inflammatory signals to significantly lower levels up to two days after infection within the ALI system. From day 3 on, also Omicron subvariants caused enhanced tissue destruction, since the viruses remain in the closed Transwell system and cannot be cleared. Studies on the virological properties of the Omicron variant, which rapidly replaced the former Delta variant in the beginning of 2022, revealed that despite its modest severity, it has a 3.3-fold higher transmissibility rate as well as an increased resistance to antiviral immunity (8, 16). These characteristics were explained by multiple amino acid substitutions in the S2 region of the Omicron spike protein that are associated with an enhanced ACE2 binding affinity (16), and an altered endocytic pathway utilizing mainly the cathepsin-dependent endocytic pathway in contrast to Delta using TMPRSS2-dependent fusion (17). In VeroE6/TMPRSS2 cells, larger syncytia formation was observed for Delta, while Omicron showed only weak syncytia formation (16). These data are in accordance with the data found here in highly differentiated, human airway epithelial cells, since infection with the Delta variant resulted in significantly higher penetration of the human airway epithelium going along with extensive tissue destruction and pore formation on 2 dpi, loss in epithelial integrity and cell death. In contrast, all Omicron subvariants tested remained apically distributed and exerted a significantly lower destruction of the pseudostratified epithelium until 2 dpi. While Meng et al. (8) described similar replication kinetics and rates of Delta and Omicron in 3D primary human nasal epithelial cells, in our 3D primary upper respiratory tract model, we found significant differences in terms of viral load and infectivity of released viruses. Delta infection resulted in high productive infection of HAE cells, whereas the replication rates and infectivity of Omicron subvariants was significantly lower. These differences can be explained by infecting with diverse infectious units or by different sampling; Meng et al. collected from the apical, i.e. air, side, while here samples were acquired from the basolateral, i.e. systemic, side to associate systemic infection rate with other factors such as inflammation, cell death, cell stress. In addition to the higher tissue destruction and viral load observed during Delta infections, higher cell death and LDH activity were detected. Previous studies reported on the pro-apoptotic activities of SARS-CoV-2 ORF3a that triggers a range of host cellular immune responses, including cellular stress and pro-inflammatory cytokine responses and induces cell death through apoptosis, necrosis and pyroptosis, thus contributing to tissue damage (12, 18). ORF3a is directly involved in activation of NLRP3 inflammasomes and pro-inflammatory cytokine induction. This in turn directly connects to the recently described ´complement-metabolism-inflammasome´ axis (19) described in immune cells. Both anaphylatoxins, C3a and C5a, have been confirmed as important drivers of NLRP3 inflammasome activation (19), and recently, exacerbating intracellular C3 mobilization as well as anaphylatoxin secretion from non-immune HAE cells following infection with SARS-CoV-2 wild type, Beta and Delta variants were described (9, 10, 15, 20). Going along with the lower pathogenicity of the Omicron subvariants in our model, here we found significantly lower complement C3 mobilization that was rather superficially distributed. In contrast, Delta-infected tissues that illustrated a deeper penetration associated with higher tissue destruction, cell death and LHD activity, showed larger C3-containing areas and C3 activation up to 30µm of depth within the tissue. The massive intracellular C3 activation was further associated with anaphylatoxin and pro-inflammatory cytokine production (9). While the Omicron subvariants only induced low-level IL-6 release from HAE cells, with the novel Omicron variants BQ1.1 and BF.7 even being less inflammatory, the Delta variant demonstrated significantly higher IL-6 levels. Limitations of this study include that cytokine and C3 expression as well as LDH release were not cross-validated by other methods. However, in earlier reports using SARS-infected tissue culture models, we extensively illustrated the association of these factors (C3, IL-6) with other proinflammatory responses (9, 20, 21). Although the main objective of our study was to compare the pathogenicity between Delta and Omicron variants, our data also revealed differences in viral loads, TEER, LDH and IL-6 expression within Omicron subvariants. While LDH and IL-6 expression were significantly higher in BA.5 compared to BQ1.1 and BF.7, viral loads and TEER were only slightly changed. In a previous study, we also demonstrated elevated viral loads in BA.5-infected HAE cultures compared to BF.7- or BQ1.1-infected tissue models (22). So far, human cohort studies investigated disease severity between Delta and Omicron waves, but only little is known about the pathogenicity between Omicron subvariants (23). Therefore, more results are needed to allow a clear statement on pathogenicity between Omicron subvariants. Recent studies on rhesus macaques and hamsters demonstrated reduced disease severity as well as cytokine and chemokine expression caused by Omicron subvariants compared to Delta (16, 24, 25). These findings are in accordance to our data using a primary human *in vitro* model. In summary, here we could shed new light on the initial pathomechanisms of human HAE cultures infected with Delta or Omicron variants, including novel subvariants such as BF.7 and BQ.1.1. Additionally, our *in vitro* findings illustrated that Delta variant penetrates deep into the pseudostratified epithelial layer and thereby initiates not only higher rates of virus release, cell death and inflammation, which was assessed by significantly higher local complement C3 and IL-6 secretion. In contrast, all Omicron subvariants tested (BA.1, BA.2, BA.5, BQ1.1, BF7) remained apically distributed. Upon focus on the novel variants BA.5, BQ1.1 and BF7, we found that in particular BQ1.1 and BF7 were associated with superficial cell death only, significantly lower systemic virus secretion and intracellular C3 mobilization and IL-6 release.

## Data availability statement

The original contributions presented in the study are included in the article/**Supplementary Material**, further inquiries can be directed to the corresponding author/s.

## Ethics statement

Written informed consent was obtained from all donors of leftover nasopharyngeal/oropharyngeal specimens and EDTA blood by the participating clinics. The Ethics Committee of the Medical University of Innsbruck (ECS1166/2020) approved the use of anonymized leftover specimens of COVID-19 patients for scientific purposes. The studies were conducted in accordance with the local legislation and institutional requirements. The participants provided their written informed consent to participate in this study.

## Author contributions

VZ: Visualization, Data curation, Investigation, Methodology, Writing – review & editing. HA: Methodology, Visualization, Writing – review & editing. A-LW: Methodology, Visualization, Writing – review & editing. GL: Investigation, Methodology, Writing – review & editing. CD: Resources, Methodology, Writing – review & editing. MM: Methodology, Resources, Writing – review & editing. AG: Methodology, Resources, Writing – review & editing. HB: Investigation, Methodology, Writing – review & editing. CL-F: Conceptualization, Supervision, Validation, Writing – review & editing. OK: Data curation, Project administration, Resources, Conceptualization, Validation, Writing – review & editing. LH: Conceptualization, Data curation, Project administration, Validation, Writing – review & editing. WP: Conceptualization, Data curation, Project administration, Writing – review & editing, Supervision, Writing – original draft, Investigation, Funding acquisition, Formal Analysis. DW: Conceptualization, Data curation, Funding acquisition, Investigation, Project administration, Supervision, Writing – original draft, Writing – review & editing, Validation, Resources, Methodology.
