# Supplementary material for: Omicron subvariants illustrate reduced respiratory tissue penetration, cell damage and inflammatory responses in human airway epithelia
Source: Front Immunol. 2023 Oct 17;14:1258268. doi: 10.3389/fimmu.2023.1258268 (PMC10616953; doi:10.3389/fimmu.2023.1258268)
Supplement: Supplementary file 1 [file DataSheet_1.docx]

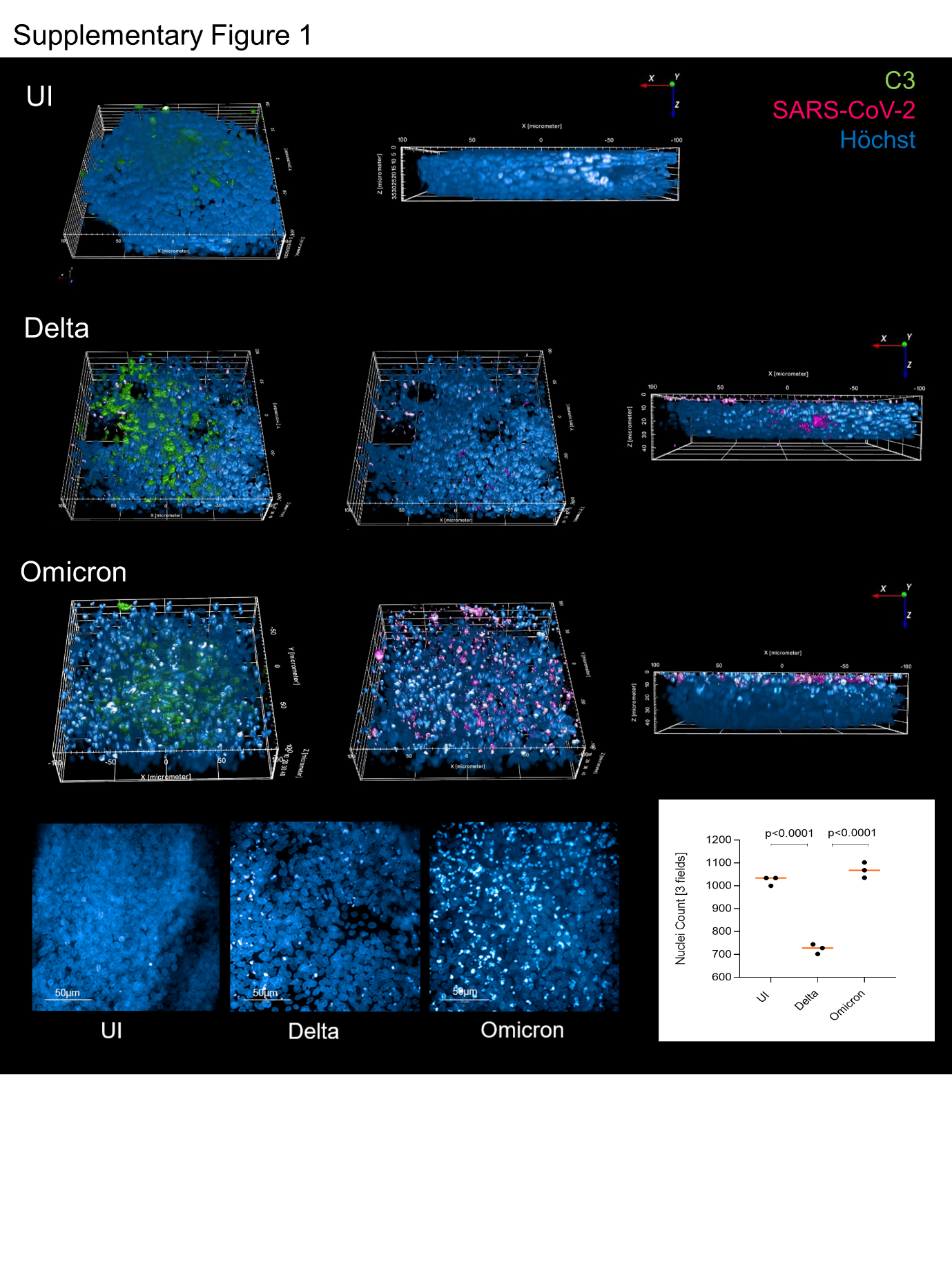


**Supplementary Figure 1. Higher complement and tissue destruction is observed in Delta- compared to Omicron-infected tissues.**

UI tissue samples exerted a compact pseudostratified layer with isolated spots of local complement C3 staining (1st panel). In contrast, Delta-infected cells illustrated multiple holes, indicating detached cells throughout the area imaged. Moreover, complement C3- areas and virus were found over the whole pseudostratified area, penetrating deep in the tissue (2nd panel). Omicron-infected cultures showed a diffuse activation of local complement C3 and only superficial virus distribution (3rd panel). Upon quantification of nuclei (4th panel), UI- and Omicron-infected cultures illustrated about 1.5-fold higher nuclei counts compared to Delta-infected HAE cultures (4th panel). Statistical significances were analyzed with GraphPad Prism software using One-way ANOVA and Tukey´s post test, means are depicted in orange.
